# The association of common infectious exposures with cognitive performance in community‐dwelling older adults

**DOI:** 10.1002/alz.70457

**Published:** 2025-10-21

**Authors:** Jackson A. Roberts, Mitchell S. V. Elkind, Minghua Liu, Stephanie Assuras, Bonnie E. Levin, Vanessa Guzman, Tatjana Rundek, Jose Gutierrez

**Affiliations:** ^1^ Department of Neurology Vagelos College of Physicians and Surgeons Columbia University New York New York USA; ^2^ Department of Neurology Massachusetts General Brigham Boston Massachusetts USA; ^3^ Department of Epidemiology Mailman School of Public Health Columbia University New York New York USA; ^4^ Department of Neurology Evelyn F. McKnight Brain Institute, Miller School of Medicine Miami Florida USA

**Keywords:** Alzheimer's disease, cognitive impairment, cytomegalovirus, dementia, Herpesviridae, memory, neuropsychological testing

## Abstract

**INTRODUCTION:**

The Northern Manhattan Study (NOMAS) previously identified that a combined infectious disease exposure index correlates with impaired cognitive performance in older adults.

**METHODS:**

We extended these findings by examining the association of serological titers of five common infectious diseases (herpes simplex virus [HSV] 1 and 2, cytomegalovirus [CMV], *Chlamydia pneumoniae*, and *Helicobacter Pylori*) with domain‐specific cognitive performance and incident cognitive impairment and dementia in 593 community‐dwelling older adults. We performed confounder‐adjusted mixed linear regression between infectious serologies and longitudinal cognitive performance.

**RESULTS:**

CMV and HSV‐2 serologies were associated with impaired executive function, whereas *C. pneumoniae* serology was associated with impaired performance on language testing. In univariate Cox proportional hazard models, CMV serologies were associated with incident cognitive impairment and dementia.

**DISCUSSION:**

CMV seropositivity may accelerate domain‐specific cognitive worsening and could confer increased risk for cognitive impairment and dementia, warranting further evaluation in observational and experimental datasets.

**Highlights:**

- Infectious exposures may contribute to neurodegeneration and risk of cognitive impairment.
- Cytomegalovirus (CMV) and herpes simplex virus 2 exposure were associated with impaired executive function.
- *Chlamydia pneumoniae* was associated with decreased performance on language testing.
- CMV may be associated with incident cognitive impairment and dementia.

## 1 BACKGROUND

The contribution of infectious disease exposures to cognitive decline and dementia has resurfaced as an area of focus in the last several years, coinciding with an interest in the impact of chronic neuroinflammation in neurodegenerative diseases.^1^ Previous work in the Northern Manhattan Study (NOMAS) identified that a composite serologic measure, referred to as the infectious burden index (IBI), of *Chlamydia pneumoniae* (*C. pneumoniae*), *Helicobacter pylori* (*H. pylori*), cytomegalovirus (CMV), herpes simplex virus 1 (HSV‐1), and herpes simplex virus 2 (HSV‐2) was associated with worse cross‐sectional cognitive performance as determined by Mini‐Mental State Exam (MMSE) and the modified Telephone Interview for Cognitive Status (TICS‐m).^2^ Similarly, the composite IBI was found to correlate with poorer baseline executive function and decline in memory scores longitudinally.^3^

Findings regarding the risk of cognitive impairment and dementia associated with infectious exposures have been heterogeneous, although evidence has suggested that some viral exposures, particularly herpesviruses, may increase risk for Alzheimer's disease (AD). HSV‐1 in particular has been associated with an increased risk of dementia, and it has been found that treatment of symptomatic infections with antivirals attenuates this association.^4^, ^5^ We additionally have found, in NOMAS participants, that HSV‐2 serologic titers correlate with decreased whole brain cortical thickness, suggesting that such infections could accelerate brain atrophy in late life.^6^ In general, herpesviruses have demonstrated the strongest links with cognitive impairment and dementia, thought to be the result of neuroinflammation resulting from chronic latent infection with cycles of reactivation resulting in activation of microglia and cytokine signaling cascades.^5^, ^7^, ^8^, ^9^ Older adults may particularly be at risk of these cycles of viral reactivation and resultant neuroinflammation due to immunosenescence in older age.^10^

In this study, we therefore sought to determine the relationship between common infectious exposures and domain‐specific cognitive performance in NOMAS participants, particularly anticipating that herpesviruses (i.e., HSV‐1 and HSV‐2 and CMV) would display the strongest negative impact on cognition. We then additionally evaluated the association between infectious serologies and incidence of cognitive impairment and dementia.

## 2 METHODS

### 2.1 Participants

Participants represented a subset of individuals in NOMAS, a longitudinal population‐based epidemiologic study of urban‐dwelling adults, as described previously.^11^ NOMAS includes participants who are randomly selected from those living in northern Manhattan with access to a home telephone, age ≥40 years, and reported to be stroke‐free at time of enrollment. A magnetic resonance imaging (MRI) sub‐study of 1290 NOMAS participants was constructed between 2003 and 2008, when stroke‐free study participants and household members ≥50 years were offered brain MRI. Of these participants, 593 had both infectious serologic testing and neuropsychological testing for evaluation of cognitive performance.

Demographic information, including age, sex, education, and race/ethnicity, was collected at the time of enrollment. Past medical history was obtained by structured questionnaire. At enrollment, laboratory testing was performed to evaluate glucose and lipid profiles. The presence of hypertension, diabetes, and hyperlipidemia was determined by self‐report and/or laboratory testing. Diabetes was defined by a fasting glucose ≥126 mg/dL. Hypercholesterolemia was defined as a total cholesterol ≥240 mg/dL. Hypertension was defined as two brachial blood pressure measurements with an average ≥140/90 mmHg on separate occasions during the study visit. Additional risk factors were recorded during annual follow‐up phone calls, which included medication use, smoking habits, and changes in diabetes or dyslipidemia status. Apolipoprotein E (*APOE*) genotyping was performed for all participants as described previously.^12^

**RESEARCH IN CONTEXT**

1. **Systematic review**: The authors utilized traditional methods to review the literature, including published manuscripts indexed in PubMed. Prior studies, including research conducted in the Northern Manhattan Study (NOMAS), have suggested that lifetime infectious exposures may impact cognitive performance and increase the risk of dementia in later life.
2. **Interpretation**: Our findings suggest that cytomegalovirus (CMV), herpes simplex virus 2, and *Chlamydia pneumoniae* exposure are associated with impaired domain‐specific cognitive performance in this cohort, and that CMV, in particular, may increase the risk of dementia.
3. **Future directions**: This study suggests the need for further research investigating the mechanisms by which common infectious exposures, particularly herpesviruses, increase the risk of cognitive impairment and dementia. Research should particularly investigate if central and peripheral inflammation associated with prior infection impact biomarkers of neurodegeneration and impact disease risk.

### 2.2 Serologic testing

Serologic testing for prior infectious disease exposures has been described previously.^13^ In brief, enzyme‐linked immunosorbent assay (ELISA) was performed using baseline visit blood samples between 1998 and 2001. Antibody titers were obtained for five pathogens: *Chlamydia pneumoniae* (*C. pneumoniae*) immunoglobulin A (IgA) (Sayvon Diagnostics, Israel), *Helicobacter pylori* (*H. pylori*) IgG and cytomegalovirus IgG (Wampole Laboratories, Princeton, New Jersey, USA), and HSV‐1 and HSV‐2 IgG (Focus Diagnostics, Cypress). Testing was performed at the Columbia University Center for Advanced Laboratory Medicine with commercially available ELISA kits in accordance with prior studies.^14^, ^15^ In accordance with our prior work,^6^, ^16^ we analyzed antibody titers in both a continuous and binary manner. Binary outcomes (i.e., seropositive or seronegative) were determined utilizing a cutoff based upon manufacturer‐recommended threshold values.

### 2.3 Cognitive assessment

Participants in the MRI sub‐study also underwent a neuropsychological battery at the time of MRI, administered by a trained research assistant, as described previously.^12^ Tests were administered in English or Spanish depending on participant preference. Neuropsychological testing was clustered into four domains based on factor analysis, with factor loading >0.3 as a requirement for test inclusion in a specific domain. Episodic memory was determined from three sub‐scores of a 12‐word, 5‐trial list‐learning task: total score, delayed recall, and delayed recognition.^17^ Semantic memory testing included the Boston Naming Test, Animal Naming, and phonemic fluency assessment with C, F, L in English speakers and F, A, S, in Spanish speakers.^18^ Processing speed was determined with the Grooved Pegboard task non‐dominant hand time and the Color Trails Test Form 1.^19^ Executive function was assessed by the difference in time to complete Color Trails Test Forms 1 and 2 and the sum of scores on the Odd‐Man‐Out subtests 2 and 4.^20^ For each test, means and SDs were calculated and within‐sample Z‐score transformation was performed. Domain‐specific Z scores were obtained by averaging Z‐scores across tests within clustered neuropsychological domains. Global cognitive performance was determined by an average of the four cognitive domains. A measure of crystallized intelligence (Gc) was constructed based on tests of vocabulary and literacy as in prior work.^12^

### 2.4 Outcome definitions

As described in prior work,^21^ a team of neuropsychologists and neurologists with expertise in dementia utilized National Institute on Aging–Alzheimer's Association (NIA‐AA) and the Diagnostic and Statistical Manual of Mental Disorders, Fifth Edition (DSM‐5), for ascertainment of diagnoses.^22^ The Cognitive Failures Questionnaire (CFQ) was administered at the time of neuropsychological testing, and the Informant Questionnaire of Cognitive Decline in the Elderly (IQCODE) was administered to family member or friend of the participant as close temporally as possible to neuropsychological testing.^23^

Neuropsychological performance was transformed into age‐ and years of education–adjusted Z‐scores derived internally at the baseline visit. The cutoffs derived from the baseline visit were subsequently applied to repeated neuropsychological tests. ^24^ Mild cognitive impairment (MCI) was diagnosed if a participant scored below –1.5 SD in 3 of 12 neuropsychological tests applied or less than –1.5 SD in a single domain but no evidence of functional impairment on the basis of (1) concern derived from the participant (based on CFQ), informant (based on IQCODE), or other witness regarding a decline in cognition; (2) decline from a prior level of performance in at least one cognitive domain; (3) lack of significant functional impairment, or (4) lack of another medical diagnosis that would explain the cognitive status.

Dementia diagnoses were determined by consensus of a multidisciplinary team with at least one neurologist or neurophysiologist, using all available information, as in prior work.^21^ This included longitudinal neuropsychological testing, assessments of functional status (IQCODE, Older Americans Resources and Services IADLs [instrumental activities of daily living], Functional Assessment Scale and/or Clinical Dementia Rating (CDR) scale score), annually performed TICS‐m, medication history, self‐reported dementia diagnoses or “cognitive failures” portion of the CDR, and review of medical charts for patients followed at our institution. Secondary assessment of longitudinal cognitive performance was performed using domain‐adjusted Z‐scores averaged across standardized scores from each visit. In analyses, “cognitive impairment” refers to individuals with MCI or dementia, whereas “dementia” refers only to those participants with an adjudicated dementia diagnosis. Individuals with dementia at the baseline visit were excluded from inclusion in the current study.

### 2.5 Statistical analysis

Multiple linear regression models, adjusted progressively for possible confounding variables, were utilized to determine the relationship between infectious disease exposures and domain‐specific cognitive performance. Model 1 was an unadjusted linear model between binary seropositivity or continuous serologic titers and domain‐specific test scores. Model 2 was adjusted for sex, age, race/ethnicity, crystallized intelligence, education, and depression. Adjustment for age is supported by prior work suggesting that seropositivity for common pathogens increases with age, and seropositivity rates have also been shown to vary by socioeconomic status, race/ethnicity, and sex.^25^, ^26^ Model 3 was adjusted for sex, age, race/ethnicity, crystallized intelligence, education, depression, hypertension, diabetes, hyperlipidemia, and smoking status. Crystallized intelligence and education were included in models as factors shown to influence neuropsychological performance independent of our exposures of interest.^27^, ^28^

We utilized Cox proportional hazard models to estimate the relationship between infectious exposures, considered as both binary and continuous variables, and incidence separately of each of cognitive impairment and dementia. Models were adjusted progressively for confounders, as follows: (1) Model 1: unadjusted univariate association between serologic titers and outcome; (2) Model 2: adjusted for sex, age, and race/ethnicity; and (3) Model 3: adjusted for sex, age, race/ethnicity, hypertension, diabetes mellitus, smoking status, and hyperlipidemia. Following Cox models, we performed a secondary analysis in which we utilized Fine–Gray subdistribution hazard models to perform competing risk Cox analysis, which account for an inability to observe the outcome of interest (i.e., cognitive impairment or dementia) due to death from another cause. Competing risk models were similarly progressively adjusted for confounding variables in the same manner as Cox models. Following analyses, a sequential Bonferroni correction (i.e., Holm procedure) for multiple comparisons was performed.^29^ The statistical analyses were carried out with SAS software, version 9.4 (SAS Institute Inc.).

## 3 RESULTS

### 3.1 Demographic characteristics

A total of 593 participants with serologic testing and neuropsychological assessment were included in the analysis. The demographic characteristics of the participants are described in Table 1. Participants were on average 71 ± 8 years of age, 39% male, and 69% Hispanic. Of those participants included in analysis, 179 (30.2%) had incident cognitive impairment and 102 (17.2%) had incident dementia diagnoses during a mean follow‐up of 7.8 years (interquartile range [IQR] 5.1–12.6 years). Twenty‐eight percent of participants (*n* = 166) died during the follow‐up period, representing a competing risk in cognitive impairment and dementia analyses. Seropositivity prevalences and mean antibody titer values are included in Table S1.

**TABLE 1:** Patient demographics and clinical data.

	Participants included (*n* = 593)	Participants excluded (*n* = 697)	*p*
Age, years, mean (SD)	71.0 (8.0)	70.4 (9.8)	0.222
Male gender, *n* (%)	231 (39.0)	279 (40.0)	0.694
Race and ethnicity, *n* (%)			0.088
Non‐Hispanic White	93 (15.7)	127 (18.2)	
Non‐Hispanic Black	92 (15.5)	131 (18.8)	
Hispanic	408 (68.8)	439 (63.0)	
Completed high school, *n* (%)	429 (72.3)	469 (67.2)	0.212
Hypertension, *n* (%)	483 (81.5)	527 (75.6)	0.011
Diabetes, *n* (%)	136 (22.9)	193 (27.7)	0.051
Hypercholesterolemia, *n* (%)	501 (84.5)	546 (78.3)	0.005
Current smoking, *n* (%)	72 (12.1)	79 (11.3)	0.653
Brain infarction, *n* (%)	108 (18.2)	129 (18.5)	0.891
Cognitive impairment^a^, *n* (%)	179 (30.2)	175 (25.8)	0.086
Dementia, *n* (%)	102 (17.2)	96 (14.2)	0.139
Competing risk event of dementia: Mortality, *n* (%)	166 (28.0)	221 (31.9)	0.138

^a^ Both mild cognitive impairment (MCI) and dementia; SD, standard deviation.

Compared to individuals in the MRI sub‐study excluded from analyses (*n* = 697) due to absence of the variables of interest, the individuals included had a higher prevalence of hypertension and hypercholesterolemia (81.5% vs 75.6% and 84.5% vs 78.3%, respectively).

### 3.2 Associations between infectious exposures and global and domain‐specific cognitive performance

In unadjusted models (Model 1), HSV‐1 (unadjusted beta: –0.200, 95% confidence interval [CI]: –0.37 to –0.03; *p* = 0.0220), CMV (unadjusted beta: –0.341, 95% CI: –0.48 to –0.20; *p* < 0.0001), and *C. pneumoniae* (unadjusted beta: –0.138, 95% CI: –0.27 to –0.01; *p* = 0.0350 seropositivity were associated with decreased global cognitive performance in unadjusted models. However, these associations attenuated to non‐significance when adjusted for confounders (Table 2). Similarly, CMV (unadjusted beta: –0.263, 95% CI: –0.46 to –0.05; *p* = 0.0149) and *C. pneumoniae* (unadjusted beta: –0.188, 95% CI: –0.36 to –0.02; *p* = 0.0314) seropositivity were associated with decreased memory scores in unadjusted models but not confounder‐adjusted models. CMV was also associated with decreased language performance in the unadjusted model (unadjusted beta: –0.352, 95% CI: –0.56 to –0.15;) = 0.0008), but not after adjustment for confounders, whereas *C. pneumoniae* was associated with decreased language scores in unadjusted and fully‐adjusted models (confounder‐adjusted beta:  –0.166, 95% CI: –0.32 to –0.01; *p* = 0.0344). For processing speed, CMV seropositivity was associated with decreased performance in the unadjusted (unadjusted beta: –0.280, 95% CI: –0.50 to –0.06; *p* = 0.0121) model only. HSV‐1 seropositivity was associated with decreased executive function in the unadjusted model only (unadjusted beta: –0.303, 95% CI: –0.53 to –0.08; *p* = 0.0079), whereas HSV‐2 (confounder‐adjusted beta: –0.188, 95% CI: –0.36 to –0.02; *p* = 0.0291) and CMV (confounder‐adjusted beta: –0.292, 95% CI: –0.51 to –0.08; *p* = 0.0074) were associated with decreased executive function in unadjusted and fully‐adjusted models. After applying the Holm procedure for multiple comparison testing, in unadjusted models (i.e., Model 1), CMV (*p* < adjusted α of 0.01) remained significantly associated with global cognition; CMV (*p* < adjusted α of 0.01) and *C. pneumoniae* (*p* < adjusted α of 0.0125) remained significantly associated with language performance; and CMV (*p* < adjusted α of 0.01), HSV‐2 (*p* < adjusted α of 0.0125), and HSV‐1 (*p* < adjusted α of 0.0167) remained significantly associated with executive function. After adjustment for multiple comparisons in fully‐adjusted models (i.e., Model 3), only CMV remained significantly associated ( < adjusted α of 0.01) with executive function.

**TABLE 2:** Association between antibody seropositivity and cognitive performance.

	Model 1		Model 2		Model 3	
Pathogen	Beta estimate (95% CI)	*p*	Beta estimate (95% CI)	*p*	Beta estimate (95% CI)	*p*
*Global cognitive performance*						
HSV‐1	−0.200 (−0.37, −0.03)	0.0220	−0.0029 (−0.18, 0.18)	0.975	0.0021 (−0.19, 0.19)	0.982
HSV‐2	−0.112 (−0.24, 0.01)	0.0761	−0.0566 (−0.18, 0.06)	0.350	−0.0524 (−0.17, 0.07)	0.401
Cytomegalovirus	−0.341 (−0.48, −0.20)	< 0.0001	−0.155 (−0.32, 0.01)	0.0631	−0.156 (−0.32, 0.01)	0.0681
*Chlamydia pneumoniae*	−0.138 (−0.27, −0.01)	0.0350	−0.092 (−0.21, 0.03)	0.134	−0.092 (−0.22, 0.03)	0.149
*Helicobacter pylori*	−0.0470 (−0.17, 0.07)	0.448	0.0291 (−0.09, 0.14)	0.622	0.0323 (−0.09, 0.15)	0.600
*Memory*						
HSV‐1	−0.0817 (−0.33, 0.17)	0.518	0.0911 (−0.16, 0.34)	0.472	0.0868 (−0.16, 0.34)	0.493
HSV‐2	−0.114 (−0.29, 0.06)	0.194	−0.0740 (−0.24, 0.09)	0.386	−0.0774 (−0.25, 0.09)	0.367
Cytomegalovirus	−0.263 (−0.47, −0.05)	0.0149	−0.122 (−0.35, 0.10)	0.290	−0.124 (−0.35, 0.10)	0.283
*C. pneumoniae*	−0.188 (−0.36, −0.02)	0.0314	−0.105 (−0.27, 0.06)	0.208	−0.105 (−0.27, 0.06)	0.207
*H. pylori*	−0.0797 (−0.25, 0.09)	0.348	0.0193 (−0.14, 0.18)	0.809	0.0251 (−0.13, 0.18)	0.755
*Language*						
HSV‐1	−0.222 (−0.12, 0.34)	0.0744	0.107 (−0.10, 0.31)	0.310	0.119 (−0.10, 0.34)	0.284
HSV‐2	−0.0678 (−0.24, 0.10)	0.440	0.0339 (−0.12, 0.19)	0.673	0.0337 (−0.13, 0.19)	0.679
Cytomegalovirus	−0.352 (−0.56, −0.15)	0.0008	−0.034 (−0.22, 0.15)	0.721	−0.0385 (−0.23, 0.15)	0.690
*C. pneumoniae*	−0.222 (−0.39, −0.05)	0.0112	−0.164 (−0.32, −0.01)	0.0337	−0.166 (−0.32, −0.01)	0.0344
*H. pylori*	−0.0823 (−0.25, 0.08)	0.333	0.0102 (−0.13, 0.16)	0.890	0.0066 (−0.14, 0.15)	0.931
*Processing speed*						
HSV‐1	−0.185 (−0.11, 0.33)	0.123	−0.0642 (−0.32, 0.19)	0.622	−0.0725 (−0.33, 0.18)	0.581
HSV‐2	−0.0584 (−0.23, 0.11)	0.499	−0.0173 (−0.18, 0.15)	0.839	−0.0192 (−0.19, 0.15)	0.821
Cytomegalovirus	−0.280 (−0.50, −0.06)	0.0121	−0.136 (−0.38, 0.11)	0.277	−0.133 (−0.38, 0.11)	0.287
*C. pneumoniae*	−0.0574 (−0.23, 0.12)	0.522	−0.0450 (−0.22, 0.13)	0.610	−0.0450 (−0.22, 0.13)	0.610
*H. pylori*	−0.0015 (−0.17, 0.16)	0.986	0.0094 (−0.15, 0.17)	0.910	0.0243 (−0.14, 0.19)	0.772
*Executive function*						
HSV‐1	−0.303 (−0.53, −0.08)	0.0079	−0.0415 (−0.27, 0.19)	0.726	−0.0374 (−0.28, 0.20)	0.758
HSV‐2	−0.251 (−0.42, −0.09)	0.0030	−0.188 (−0.35, −0.02)	0.0261	−0.188 (−0.36, −0.02)	0.0291
Cytomegalovirus	−0.504 (−0.70, −0.31)	< 0.0001	−0.289 (−0.50, −0.08)	0.0074	−0.292 (−0.51, −0.08)	0.0074
*C. pneumoniae*	−0.073 (−0.25, 0.10)	0.410	−0.059 (−0.23, 0.11)	0.500	−0.059 (−0.23, 0.11)	0.500
*H. pylori*	−0.0045 (−0.17, 0.17)	0.959	0.104 (−0.07, 0.28)	0.240	0.102 (−0.08, 0.28)	0.258

Model 1: unadjusted univariate model between seropositivity and the outcome.

Model 2: adjusted for sex, age, ethnicity, crystallized intelligence, education, and depression.

Model 3: adjusted for age, sex, ethnicity, crystallized intelligence, education, depression, hypertension, diabetes, hyperlipidemia, and smoking status.

We additionally determined the association between continuous serologic titers for each pathogen and cognitive performance in each domain (Table S2). Results[Table alz70457-tbl-0003] were overall consistent with the primary analysis of seropositivity in association with cognitive performance. In confounder‐adjusted models (i.e., Model 3), continuous *C. pneumoniae* titers were associated with global cognitive performance (confounder‐adjusted beta: –0.031, 95% CI: –0.054 to –0.009; *p* = 0.007), memory (confounder‐adjusted beta: –0.044, 95% CI: –0.082 to –0.006; *p* = 0.023), and language (confounder‐adjusted beta: –0.041, 95% CI: –0.074 to –0.008; *p* = 0.016). In fully‐adjusted models, CMV titers were associated with decreased executive function (confounder‐adjusted beta: –0.076, 95% CI: –0.144 to –0.009; *p* = 0.027). In unadjusted models, CMV titers were associated with decreased global cognitive performance (unadjusted beta: –0.069, 95% CI: –0.130 to –0.044; *p* < 0.0001), language (unadjusted beta: –0.105, 95% CI: –0.166 to –0.044; *p* = 0.001), and processing speed (unadjusted beta: –0.091, 95% CI: –0.154 to –0.029; *p* = 0.004). In unadjusted models only, HSV‐2 titers were associated with decreased global cognitive performance (unadjusted beta: –0.028, 95% CI: –0.050 to –0.006; *p* = 0.012), and HSV‐2 was associated with decreased executive function (confounder‐adjusted beta: –0.035, 95% CI: –0.067 to –0.004; *p* = 0.027) in confounder‐adjusted models. HSV‐1 was also associated with global cognitive performance (unadjusted beta: –0.037, 95% CI: –0.069 to –0.005; *p* = 0.024) and executive function (unadjusted beta: –0.50, 95% CI: –0.079 to –0.019; *p* = 0.028) in unadjusted models only. *H. pylori* titers were associated with increased processing speed in confounder‐adjusted models (confounder‐adjusted beta: 0.002, 95% CI: 0.000–0.003; *p* = 0.034). After adjustment for multiple comparisons in unadjusted models (i.e., Model 1), CMV, *C. pneumoniae*, HSV‐1, and HSV‐2 remained significantly associated with global cognitive performance; *C. pneumoniae* remained associated with memory; CMV and *C. pneumoniae* remained associated with language performance; CMV remained associated with processing speed; and CMV, HSV‐2, and HSV‐1 remained associated with executive function. In confounder‐adjusted models (i.e., Model 3), only *C. pneumoniae* remained significantly associated with global cognitive performance after multiple comparison correction.

### 3.3 Associations of infectious exposures with incident mild cognitive impairment

CMV seropositivity was associated with an increased risk of MCI (unadjusted hazard ratio [HR] = 1.22, 95% CI: 1.09–1.36; *p* = 0.0005), but the significance did not persist with adjustment for confounders (Table 3). Similarly, CMV titers considered as continuous variables were associated with an increased risk of MCI in the univariate analysis only (unadjusted HR = 1.67, 95% CI: 1.06–2.64; *p* = 0.027). These results persisted in competing risk models, in which CMV continuous titers and seropositivity were associated with an increased risk of MCI in univariate models only (Table S3). After correction for multiple comparisons, only CMV seropositivity but not continuous titers remained associated with incident cognitive impairment in unadjusted models.

**TABLE 3 alz70457-tbl-0003:** Association of pathogen serologic titers with incident cognitive impairment.

	Model 1		Model 2		Model 3	
Pathogen	Hazard ratio	*p*	Hazard ratio	*p*	Hazard ratio	*p*
*Binary (seropositive vs seronegative)*						
HSV‐1	0.96 (0.88–1.04)	0.282	0.95 (0.87–1.04)	0.296	0.95 (0.87–1.03)	0.203
HSV‐2	1.04 (0.99–1.10)	0.126	1.04 (0.99–1.11)	0.147	1.04 (0.99–1.11)	0.145
Cytomegalovirus	1.22 (1.09–1.36)	0.0005	1.11 (0.99–1.25)	0.083	1.10 (0.98–1.24)	0.121
*Chlamydia pneumoniae*	1.00 (0.92–1.08)	0.926	1.03 (0.96–1.11)	0.394	1.03 (0.96–1.10)	0.466
*Helicobacter pylori*	1.00 (1.00–1.01)	0.196	1.00 (1.00–1.00)	0.934	1.00 (1.00–1.00)	0.962
*Continuous serologic titers*						
HSV‐1	0.98 (0.63–1.52)	0.926	1.02 (0.63–1.62)	0.960	1.05 (0.66–1.68)	0.845
HSV‐2	1.33 (0.98–1.81)	0.067	1.34 (0.97–1.85)	0.076	1.35 (0.98–1.87)	0.703
Cytomegalovirus	1.67 (1.06–2.64)	0.027	1.47 (0.88–2.47)	0.143	1.50 (0.89–2.53)	0.125
*C. pneumoniae*	0.79 (0.59–1.07)	0.125	0.94 (0.69–1.27)	0.681	0.92 (0.68–1.26)	0.616
*H. pylori*	1.10 (0.82–1.49)	0.522	1.15 (0.84–1.56)	0.383	1.15 (0.85–1.57)	0.363

Model 1: unadjusted univariate association.

Model 2: adjusted for sex, age, and ethnicity.

Model 3: adjusted for sex, age, ethnicity, hypertension, diabetes mellitus, smoking status, and hyperlipidemia.

### 3.4 Associations of infectious exposures with incident dementia

In both binary and continuous analyses, CMV was associated with an increased risk of incident dementia (unadjusted HR = 1.18, 95% CI: 1.02–1.37; *p* = 0.024 and unadjusted HR: 2.04, 95% CI: 1.06–3.93; *p* = 0.032, respectively) in univariate models only (Table S4). In competing risk models, continuous titers of CMV were associated with incident dementia in the univariate model only (Table S5). After correction for multiple comparisons, CMV seropositivity but not continuous titers remained associated with incident dementia impairment in unadjusted models.

## 4 DISCUSSION

In this study, we sought to determine the relationship between common infectious disease serologies and cognitive performance and incident MCI and dementia in community‐dwelling adults who were disease‐free at the time of enrollment. We found that CMV seropositivity was associated with reduced executive function across confounder‐adjusted models, whereas *C. pneumoniae* was associated with decreased language performance and HSV‐2 was associated with decreased executive function across all models. In addition, CMV was associated with incident MCI and dementia in univariate analyses, although the significance of these associations did not persist with covariate adjustment. Taken together, these findings suggest that CMV seropositivity may negatively impact cognitive performance in a domain‐specific manner and could potentially confer risk for the development of MCI and dementia, although the statistical significance of this latter association should be interpreted as preliminary.

CMV is a betaherpesvirus ubiquitous in human populations, with seropositivity estimates ranging from 40% to 80%.^30^ Of interest, CMV IgG has previously been identified to be associated with reduced cognitive functioning even among younger individuals, with a mean age of 32.8 years in one study.^31^ In a more comparable study of community‐dwelling older adults, CMV serological titers were similarly associated with reduced cognitive function, particularly among individuals with lower educational attainment.^32^ Compared to that prior study, our cohort has a lower level of secondary education completion, which may partially explain the persistence of this association across adjusted models. It is notable that CMV seropositivity is significantly more prevalent in non‐Hispanic Black and Hispanic adults as compared to non‐Hispanic White individuals,^33^ groups that also experience higher rates of cognitive decline and dementia.^34^, ^35^ A comparable study of community‐dwelling Hispanic older adults also identified that CMV, but not HSV‐1, seropositivity was associated with more rapid decline in MMSE performance.^36^ Others have also found that higher CMV serologic titers correspond with lower cognitive performance, although in the present study such an effect was not reliably observed after confounder adjustment.^31^, ^37^ With respect to the development of dementia, CMV seropositivity has been shown previously to increase the risk of developing AD in prior cohort and case‐control studies, both in combination with and independent of concomitant HSV‐1 exposure.^38^, ^39^ We similarly identified an increased risk of MCI and dementia in our study, although only in univariate models. Others have identified a possible synergistic effect of CMV and HSV‐1 co‐exposure on global cognitive performance,^40^ a finding previously corroborated in the NOMAS cohort, in which a combined IBI was associated with MMSE.^2^

The mechanisms underlying these findings remain under investigation; however, similar to the alphaherpesviruses, latency and reactivation throughout the life course are thought to drive this potential risk.^41^ CMV infection may undergo cycles of reactivation, although rare in the immunocompetent, leading to peripheral inflammation that has been associated with brain atrophy and cognitive decline.^42^ CMV infection also necessitates the devotion of significant host immune system resources to maintain viral suppression, which may contribute to early immune senescence and has been associated with impaired vascular health.^43^, ^44^ Indeed, one study identified that CMV seropositivity was associated with an inversion of the CD:CD8 ratio, a biomarker of immune senescence and chronic inflammation, that was associated with cognitive and functional impairment.^45^, ^46^ These associational findings therefore suggest the need for further investigation elucidating the mechanisms by which CMV exposure may drive risk for cognitive impairment and dementia, focused particularly on peripheral inflammation and immunosenescence in older adults.

Although neither was associated with incidence of MCI or dementia, HSV‐2 was associated with executive functioning, and *C. pneumoniae* was associated with language in a domain‐specific manner. We previously identified that HSV‐2 was associated with cortical thickness in the NOMAS cohort,^6^ which has been associated previously with executive dysfunction in individuals with Alzheimer's disease (AD).^47^ Although HSV‐1 has more classically been considered in association with an increased risk of AD,^48^, ^49^ it has been found that symptomatic non‐serotyped herpes simplex virus infections increase the risk of incident dementia, and risk is reduced with antiviral treatment.^4^ It is unclear whether this risk is mediated by peripheral or central inflammation, although even mild and asymptomatic cases of herpes simplex encephalitis have been observed to undergo periods of viral latency and reactivation that could contribute to chronic inflammation.^50^

*C. pneumoniae* is a common respiratory bacterium that has been identified in post‐mortem AD brain tissue^51^, ^52^ and was weakly associated with whole brain cortical thickness in prior work.^6^ In this study, *C. pneumoniae* was associated with decreased performance on language testing, which is known as an early feature of dementia that worsens with disease progression.^53^ In one Taiwanese national cohort study, treatment of *C. pneumonia* infections with antibiotics was found to decrease the risk for AD development,^54^ and in vitro work has identified the capacity of this bacterium to stimulate neuroinflammatory and amyloid production pathways in astrocytes.^55^ *C. pneumoniae* has also been demonstrated to be pro‐atherogenic, possibly recruiting inflammatory T lymphocytes to the site of atheromas, suggesting that exposure to the pathogen may impact cognitive functioning by impacting vascular health.^56^

Our study is limited by the use of serologic titers, which provide markers of exposure intensity rather than pathogen activity, thereby precluding an assessment of the impact of active or reactivating infection in this study. For *C. pneumoniae*, however, we used IgA titers, which are thought to represent ongoing infection and mucosal immunity. We also did not collect information regarding symptomatic episodes secondary to infection and are unable to assess the impact of potential antiviral or antibiotic therapy received by participants. Due to the original data collection procedures, there was also a gap between the original collection of serologic titers and cognitive testing during which individuals in the study could possibly seroconvert as a result of novel infectious exposures. This would result in the potential misclassification of such individuals as seronegative and may potentially contribute to weakening of some of the associations analyzed. In addition, the study is limited by sample size, particularly the low number of individuals with cognitive impairment and dementia, which limits the statistical power of the analyses performed. As a result, several of the associations explored in this study do not persist after correction for multiple comparisons and thus warrant confirmation in larger cohort studies. Despite these limitations, the study is notable for its inclusion of a community‐dwelling population with sociodemographic diversity more reflective of the general population than many studies of dementia. We additionally controlled for multiple confounding comorbidities and sociodemographic variables that may impact the relationship between infectious exposures and cognitive performance.

Taken together, we provide evidence that CMV exposure is associated with decreased executive function and possibly increases risk for cognitive impairment and dementia, adding to a growing body of evidence that common infectious exposures may contribute to cognitive aging and dementia in late life. We also identify that HSV‐2 and *C. pneumoniae* exposures were associated with domain‐specific cognitive performance. These preliminary associations warrant exploration in future work to explore the role of central and peripheral inflammation and confirm results in larger studies with robust neuropsychological characterization of participants.

## CONFLICT OF INTEREST STATEMENT

The authors report no personal or financial interests relevant to this study.

## Supporting information



Supporting Information

Supporting Information
