# Supplementary material for: The association of common infectious exposures with cognitive performance in community‐dwelling older adults
Source: Alzheimers Dement. 2025 Oct 21;21(10):e70457. doi: 10.1002/alz.70457 (PMC12538643; doi:10.1002/alz.70457)
Supplement: Supplementary file 2 — Supporting Information [file ALZ-21-e70457-s002.docx]

**SUPPLEMENTARY TABLES**

**Supplementary Table 1. Seropositivity and mean antibody titers for infectious exposures.**

|  | Seropositivity, % | Antibody Titers (mean ± SD) |
| --- | --- | --- |
| Herpes simplex virus 1 | 512 (86.3) | 3.9 ± 1.7 |
| Herpes simplex virus 2 | 368 (62.1) | 3.2 ± 2.7 |
| Cytomegalovirus | 495 (83.5) | 2.6 ± 1.3 |
| Chlamydia pneumoniae | 373 (62.9) | 2.1 ± 2.0 |
| Helicobacter pylori | 337 (56.8) | 34.2 ± 38.1 |

**Supplementary Table 2. Association between continuous antibody titers and cognitive performance**

|  | **Model 1** | | **Model 2** | | **Model 3** | |
| --- | --- | --- | --- | --- | --- | --- |
| **Pathogen** | Beta estimate (lower 95%, upper 95%) | P-value | Beta estimate (lower 95%, upper 95%) | P-value | Beta estimate (lower 95%, upper 95%) | P-value |
| ***Global cognitive performance*** | | | | | | |
| **HSV-1** | **-0.037 (-0.069, -0.005)** | **0.024** | -0.004 (-0.036, 0.029) | 0.825 | -0.0004 (-0.035, 0.034) | 0.983 |
| **HSV-2** | **-0.028 (-0.050, -0.006)** | **0.012** | -0.014 (-0.035, 0.008) | 0.202 | -0.014 (-0.037, 0.008) | 0.214 |
| **Cytomegalovirus** | **-0.069 (-0.130, -0.044)** | **<0.0001** | -0.0432 (-0.088, 0.002) | 0.0596 | -0.035 (-0.082, 0.012) | 0.141 |
| ***C. pneumoniae*** | **-0.038 (-0.063, -0.013)** | **0.0032** | **-0.027 (-0.048, -0.006)** | **0.0104** | **-0.031 (-0.054, -0.009)** | **0.007** |
| *H. pylori* | -0.0003 (-0.002, 0.001) | 0.659 | 0.0005 (-0.001, 0.002) | 0.443 | 0.0004 (-0.001, 0.002) | 0.523 |
| ***Memory*** | | | | | | |
| HSV-1 | -0.025 (-0.071, 0.021) | 0.281 | 0.005 (-0.040, 0.051) | 0.821 | 0.006 (-0.039, 0.052) | 0.792 |
| HSV-2 | -0.028 (-0.059, 0.003) | 0.075 | -0.019 (-0.050, 0.012) | 0.221 | -0.020 (-0.051, 0.011) | 0.210 |
| Cytomegalovirus | -0.051 (-0.112, 0.010) | 0.102 | -0.019 (-0.080, 0.041) | 0.530 | -0.017 (-0.078, 0.045) | 0.595 |
| ***C. pneumoniae*** | **-0.066 (-0.105, -0.027)** | **0.001** | **-0.043 (-0.081, -0.006)** | **0.024** | **-0.044 (-0.082, -0.006)** | **0.023** |
| *H. pylori* | -0.001 (-0.003, 0.001) | 0.194 | -0.0002 (-0.002, 0.001) | 0.805 | -0.0002 (-0.002, 0.001) | 0.857 |
| ***Language*** | | | | | | |
| HSV-1 | -0.040 (-0.086, 0.006) | 0.088 | 0.009 (-0.031, 0.049) | 0.648 | 0.011 (-0.031, 0.053) | 0.608 |
| HSV-2 | -0.023 (-0.053, 0.007) | 0.140 | 0.004 (-0.025, 0.032) | 0.809 | 0.002 (-0.027, 0.031) | 0.904 |
| **Cytomegalovirus** | **-0.105 (-0.166, -0.044)** | **0.001** | -0.024 (-0.079, 0.031) | 0.395 | -0.022 (-0.079, 0.034) | 0.435 |
| ***C. pneumoniae*** | **-0.055 (-0.097, -0.013)** | **0.010** | **-0.038 (-0.068, -0.007)** | **0.017** | **-0.041 (-0.074, -0.008)** | **0.016** |
| *H. pylori* | -0.0001 (-0.002, 0.001) | 0.935 | 0.001 (-0.001, 0.002) | 0.491 | 0.0004 (-0.001, 0.002) | 0.571 |
| ***Processing Speed*** | | | | | | |
| HSV-1 | -0.030 (-0.076, 0.015) | 0.190 | -0.015 (-0.063, 0.033) | 0.536 | -0.009 (-0.057, 0.040) | 0.725 |
| HSV-2 | -0.020 (-0.051, 0.011) | 0.214 | -0.008 (-0.038, 0.023) | 0.628 | -0.008 (-0.039, 0.023) | 0.630 |
| **Cytomegalovirus** | **-0.091 (-0.154, -0.029)** | **0.004** | -0.054 (-0.119, 0.012) | 0.107 | -0.038 (-0.104, 0.027) | 0.253 |
| *C. pneumoniae* | -0.008 (-0.041, 0.026) | 0.665 | -0.002 (-0.034, 0.030) | 0.893 | -0.006 (-0.038, 0.026) | 0.718 |
| *H. pylori* | 0.001 (0.000, 0.003) | 0.055 | 0.002 (0.000, 0.0003) | 0.042 | 0.002 (0.000, 0.003) | 0.034 |
| ***Executive Function*** | | | | | | |
| HSV-1 | **-0.050 (-0.094, -0.006)** | **0.028** | -0.007 (-0.053, 0.040) | 0.781 | -0.004 (-0.052, 0.043) | 0.857 |
| **HSV-2** | **-0.049 (-0.079, -0.019)** | **0.001** | **-0.034 (-0.065, -0.004)** | **0.026** | **-0.035 (-0.067, -0.004)** | **0.027** |
| **Cytomegalovirus** | **-0.135 (-0.195, -0.075)** | **<0.0001** | **-0.080 (-0.145, -0.015)** | **0.016** | **-0.076 (-0.144, -0.009)** | **0.027** |
| *C. pneumoniae* | -0.032 (-0.068, 0.005) | 0.086 | -0.030 (-0.066, 0.007) | 0.115 | -0.033 (-0.070, 0.004) | 0.081 |
| *H. pylori* | 0.0001 (-0.002, 0.002) | 0.927 | 0.001 (-0.001, 0.003) | 0.328 | 0.001 (-0.001, 0.003) | 0.368 |

Model 1: unadjusted univariate model between seropositivity and the outcome

Model 2: adjusted for sex, age, ethnicity, crystallized intelligence, education, and depression

Model 3: adjusted for age, sex, ethnicity, crystallized intelligence, education, depression, hypertension, diabetes, hyperlipidemia, and smoking status

**Supplementary Table 3. Competing risk Cox models evaluating the association of pathogen serologic titers with cognitive impairment.**

|  | **Model 1** | | **Model 2** | | **Model 3** | |
| --- | --- | --- | --- | --- | --- | --- |
| **Pathogen** | Hazard Ratio | p-value | Hazard Ratio | p-value | Hazard Ratio | p-value |
| ***Binary (seropositive vs seronegative)*** | | | | | | |
| HSV-1 | 0.98 (0.90-1.07) | 0.574 | 0.97 (0.89-1.06) | 0.453 | 0.97 (0.89-1.05) | 0.410 |
| HSV-2 | 1.03 (0.98-1.09) | 0.263 | 1.02 (0.96-1.08) | 0.567 | 1.02 (0.96-1.08) | 0.477 |
| Cytomegalovirus | **1.14 (1.03-1.26)** | **0.009** | 1.05 (0.94-1.18) | 0.364 | 1.04 (0.93-1.16) | 0.528 |
| *C. pneumoniae* | 0.99 (0.91-1.07) | 0.716 | 1.01 (0.94-1.09) | 0.800 | 1.01 (0.93-1.09) | 0.871 |
| *H. pylori* | 1.00 (1.00-1.01) | 0.197 | 1.00 (1.00-1.00) | 0.557 | 1.00 (1.00-1.00) | 0.603 |
| ***Continuous Serologic Titers*** | | | | | | |
| HSV-1 | 1.09 (0.69-1.71) | 0.724 | 1.00 (0.61-1.65) | 0.987 | 1.03 (0.63-1.70) | 0.896 |
| HSV-2 | 1.25 (0.93-1.69) | 0.148 | 1.20 (0.86-1.67) | 0.278 | 1.22 (0.87-1.70) | 0.242 |
| Cytomegalovirus | **1.63 (1.04-2.57)** | **0.035** | 1.43 (0.86-2.40) | 0.169 | 1.38 (0.82-2.34) | 0.227 |
| *C. pneumoniae* | 0.82 (0.62-1.10) | 0.190 | 0.93 (0.68-1.26) | 0.628 | 0.93 (0.68-1.27) | 0.634 |
| *H. pylori* | 1.07 (0.80-1.44) | 0.632 | 1.08 (0.80-1.46) | 0.609 | 1.07 (0.79-1.44) | 0.659 |

Model 1: unadjusted univariate association

Model 2: adjusted for sex, age, and ethnicity

Model 3: adjusted for sex, age, ethnicity, hypertension, diabetes mellitus, smoking status, and hyperlipidemia

**Supplementary Table 4. Cox models evaluating the association of pathogen serologic titers with incident dementia.**

|  | **Model 1** | | **Model 2** | | **Model 3** | |
| --- | --- | --- | --- | --- | --- | --- |
| **Pathogen** | Hazard Ratio | p-value | Hazard Ratio | p-value | Hazard Ratio | p-value |
| ***Binary (seropositive vs seronegative)*** | | | | | | |
| HSV-1 | 0.98 (0.88-1.09) | 0.704 | 0.96 (0.85-1.08) | 0.475 | 0.95 (0.85-1.07) | 0.405 |
| HSV-2 | 1.05 (0.98-1.13) | 0.198 | 1.05 (0.98-1.14) | 0.190 | 1.06 (0.98-1.14) | 0.166 |
| Cytomegalovirus | **1.18 (1.02-1.37)** | **0.024** | 1.04 (0.89-1.22) | 0.607 | 1.03 (0.88-1.21) | 0.703 |
| *C. pneumoniae* | 0.99 (0.89-1.10) | 0.804 | 1.04 (0.94-1.14) | 0.470 | 1.04 (0.95-1.14) | 0.443 |
| *H. pylori* | 1.00 (1.00-1.01) | 0.294 | 1.00 (0.99-1.00) | 0.515 | 1.00 (0.99-1.00) | 0.540 |
| ***Continuous Serologic Titers*** | | | | | | |
| HSV-1 | 1.09 (0.61-1.95) | 0.783 | 1.05 (0.56-1.98) | 0.884 | 1.12 (0.59-2.13) | 0.724 |
| HSV-2 | 1.32 (0.88-1.98) | 0.183 | 1.43 (0.93-2.20) | 0.108 | 1.48 (0.95-2.30) | 0.084 |
| Cytomegalovirus | **2.04 (1.06-3.93)** | **0.032** | 1.81 (0.85-3.83) | 0.123 | 1.92 (0.90-4.10) | 0.093 |
| *C. pneumoniae* | 0.70 (0.48-1.04) | 0.078 | 0.86 (0.58-1.28) | 0.468 | 0.86 (0.58-1.29) | 0.464 |
| *H. pylori* | 1.03 (0.70-1.52) | 0.887 | 0.99 (0.66-1.48) | 0.942 | 1.01 (0.67-1.51) | 0.977 |

Model 1: unadjusted univariate association

Model 2: adjusted for sex, age, and ethnicity

Model 3: adjusted for sex, age, ethnicity, hypertension, diabetes mellitus, smoking status, and hyperlipidemia

**Supplementary Table 5. Competing risk Cox models evaluating the association of pathogen serologic titers with incident dementia.**

|  | **Model 1** | | **Model 2** | | **Model 3** | |
| --- | --- | --- | --- | --- | --- | --- |
| **Pathogen** | Hazard Ratio | p-value | Hazard Ratio | p-value | Hazard Ratio | p-value |
| ***Binary (seropositive vs seronegative)*** | | | | | | |
| HSV-1 | 1.00 (0.90-1.11) | 0.974 | 0.97 (0.86-1.09) | 0.614 | 0.97 (0.86-1.09) | 0.578 |
| HSV-2 | 1.04 (0.96-1.11) | 0.345 | 1.03 (0.96-1.11) | 0.434 | 1.04 (0.96-1.12) | 0.367 |
| Cytomegalovirus | 1.09 (0.96-1.23) | 0.200 | 0.96 (0.83-1.11) | 0.560 | 0.94 (0.81-1.09) | 0.414 |
| *C. pneumoniae* | 0.97 (0.86-1.08) | 0.553 | 0.99 (0.90-1.09) | 0.869 | 1.00 (0.91-1.09) | 0.921 |
| *H. pylori* | 1.00 (1.00-1.01) | 0.312 | 1.00 (1.00-1.01) | 0.815 | 1.00 (1.00-1.01) | 0.856 |
| ***Continuous Serologic Titers*** | | | | | | |
| HSV-1 | 1.15 (0.64-2.07) | 0.634 | 0.98 (0.51-1.90) | 0.949 | 1.00 (0.52-1.91) | 0.995 |
| HSV-2 | 1.24 (0.83-1.84) | 0.299 | 1.30 (0.83-2.04) | 0.249 | 1.35 (0.85-2.16) | 0.203 |
| Cytomegalovirus | **1.93 (1.01-3.69)** | **0.048** | 1.69 (0.79-3.61) | 0.176 | 1.62 (0.75-3.53) | 0.223 |
| *C. pneumoniae* | 0.74 (0.50-1.09) | 0.125 | 0.88 (0.58-1.34) | 0.551 | 0.87 (0.57-1.33) | 0.528 |
| *H. pylori* | 1.00 (0.68-1.47) | 0.990 | 0.97 (0.65-1.43) | 0.859 | 0.96 (0.64-1.42) | 0.821 |

Model 1: unadjusted univariate association

Model 2: adjusted for sex, age, and ethnicity

Model 3: adjusted for sex, age, ethnicity, hypertension, diabetes mellitus, smoking status, and hyperlipidemia
